# Comparing Time Efficiency of Sprint vs. High-Intensity Interval Training in Reducing Abdominal Visceral Fat in Obese Young Women: A Randomized, Controlled Trial

**DOI:** 10.3389/fphys.2018.01048

**Published:** 2018-08-03

**Authors:** Tomas K. Tong, Haifeng Zhang, Hongru Shi, Yang Liu, Jingwen Ai, Jinlei Nie, Zhaowei Kong

**Affiliations:** ^1^Department of Physical Education, Hong Kong Baptist University, Kowloon, Hong Kong; ^2^Physical Education College, Hebei Normal University, Shijiazhuang, China; ^3^Provincial Key Lab of Measurement and Evaluation in Human Movement and Bio-Information, Hebei Normal University, Shijazhuang, China; ^4^School of Physical Education and Sports, Macao Polytechnic Institute, Macau, Macau; ^5^Faculty of Education, University of Macau, Taipa, Macau

**Keywords:** high-intensity interval training, intervention, obesity, abdominal fat, visceral fat area

## Abstract

**Introduction:** High-intensity interval training (HIIT) is an emerging lifestyle intervention strategy for controlling obesity. HIIT consisted of brief all-out supramaximal sprint intervals was termed as sprint interval training (SIT). This study was designed to examine the time-efficient characteristics of SIT in reducing abdominal visceral fat.

**Methods:** A randomized controlled trial was conducted to compare the specific adaptations of SIT (80 × 6 s all-out cycle sprints interspersed with 9 s passive recovery) with those resulting from a HIIT regimen with training volume relatively higher (repeated 4 min bouts of cycling at 90% $\dot{\text{V}}$O_2*max*_ alternated with 3 min rest, until the work of 400KJ was achieved), and with those of nonexercising control counterparts (CON). Forty-six obese young women (body fat percentage ≥30) received either SIT (*n* = 16), HIIT (*n* = 16), or no training (*n* = 14), 3–4 sessions per week, for 12 weeks. The abdominal visceral fat area (AVFA) and abdominal subcutaneous fat area (ASFA) of the participants were measured through computed tomography scans pre-intervention and post-intervention. Total fat mass and the fat mass of the android, gynoid, and trunk regions were assessed through dual-energy X-ray absorptiometry.

**Results:** Following the intervention, abdominal visceral and subcutaneous fat were reduced markedly (*p* < 0.05). The reduction in AVFA (−6.31, −9.7 cm^2^) was not different between SIT and HIIT (*p* > 0.05), while the reduction in ASFA (−17.4, −40.7 cm^2^) in SIT was less than that in HIIT (*p* < 0.05). Less reduction in the fat mass of the trunk (−1.2, −2.0 kg) region was also found in SIT, while the reductions in fat percentage (−1.9%, −2.0%), total fat mass (−2.0, −2.8 kg), and fat mass of the android (−0.2, −0.2 kg), and gynoid (−0.4, −0.3 kg) regions did not differ between the two regimes (*p* > 0.05). In contrast, the increase in $\dot{\text{V}}$O_2*max*_ was significant greater following the SIT than HIIT (*p* < 0.01). No variable changed in CON.

**Conclusion:** Such findings suggest that the lower training load and exercise time commitments of the SIT regime could optimize the time-efficiency advantage of the traditional HIIT, facilitating the abdominal visceral fat reduction in obese young women.

## Introduction

In recent years, emerging evidences suggest that the HIIT could induce favorable adaptations in the control of “metabolic obesity,” in reference to intra-abdominal visceral fat accumulation (Zhang et al., 2015, 2017; Maillard et al., 2016, 2018). The HIIT regimen, which requires less commitment by participants in terms of time compared with those of prolonged aerobic-type exercise, is assumed to be more advantageous than continuous endurance training in developing time-efficient lifestyle intervention strategies for controlling obesity-related complications (Nie et al., 2012). However, only a limited number of studies have systematically examined the impact of HIIT protocols on abdominal visceral fat reduction (Maillard et al., 2018). A time-efficient HIIT protocol for reducing the specific abdominal fat remains elusive.

Recently, the dose-response effect of HIIT on visceral fat reduction was examined in obese women by increasing the repetitions of high-intensity exercise in each training session (Zhang et al., 2015, 2017). The increased training volume did not correspondingly induce greater visceral fat loss, implying that the training volume of the HIIT may not be a relevant variable for modifying abdominal visceral fat storage (Zhang et al., 2017). On the other hand, the facilitation effects of high-intensity exercise, in comparison to low-intensity exercise, on abdominal visceral fat reduction have been demonstrated in obese women participated in endurance exercise training, and was partly attributed to the greater negative energy balance, and the potential increase in lipolytic hormone secretion (Irving et al., 2008). Specifically, the lipolytic hormone of catecholamines promote lipolysis through β_3_-adrenoceptors, which are more common in abdominal fat than in subcutaneous fat (Ibrahim, 2010). In light of the previous findings, it was reasonable to postulate that the exercise intensity of the HIIT protocol, rather than the training volume, is the foundation of eliminating excess visceral fat in obese individual.

High-intensity interval training consisted of brief (<30 s) all-out supramaximal (>100% $\dot{\text{V}}$O_2*max*_) sprint intervals was termed as SIT (Weston et al., 2014). Previous SIT regimens have been shown effective to both healthy young women and overweight/obese young men for fat loss and energetic adaptations (Trapp et al., 2008; Heydari et al., 2012; Jabbour et al., 2015). However, whether the SIT regimen was efficacious in healthy obese population to reduce the visceral fat in an efficient manner has not been illustrated. The purposes of this study were to examine (1) the effects of 12 week SIT regimen (80 × 6 s all-out cycle sprints interspersed with 9 s passive recovery per session, 3–4 sessions per week) on the reduction of whole-body, abdominal visceral, and abdominal subcutaneous fat, as assessed through DEXA and computed tomography (CT), in premenopausal young obese Chinese women; and (2) the time-efficiency in visceral fat loss induced by the SIT vs the traditional HIIT. A randomized controlled trial was conducted to compare the specific SIT adaptations with those resulting from an existing prolonged submaximal HIIT regime (interval exercises at an intensity of 90% $\dot{\text{V}}$O_2*max*_, with accumulated exercise time of >30 min in each session) for visceral fat loss (Zhang et al., 2017), and with those of nonexercising control counterparts (CON). The intensity of the SIT and HIIT were revealed by the mean power output of the exercise on a cycle ergometer; while the internal training load imposed on participants during the two regimes was quantified by the HR-based method of TRIMP proposed by Banister (1991). As equivalent exercise-induced energy expenditure in participants was not likely to be deliberately manipulated between the SIT and HIIT groups in the present study, an explicit prolonged HIIT regimen with work done of 400 KJ was adopted. According to our pilot trials (data not shown), higher energy expenditure elicited by a single session of the HIIT in comparison to that of the SIT was assumed. This intended to avoid the unfair comparison in the resultant fat loss between the two training programs due to the advantage of greater negative energy balance resulting from the higher exercise intensity taken by the SIT group. It was hypothesized that comparable reduction in visceral fat and alterations in other body composition parameters would be resulted from the time-efficient SIT regime and the prolonged submaximal HIIT program following 12 week intervention period.

## Materials and Methods

### Participants

**FIGURE 1:**
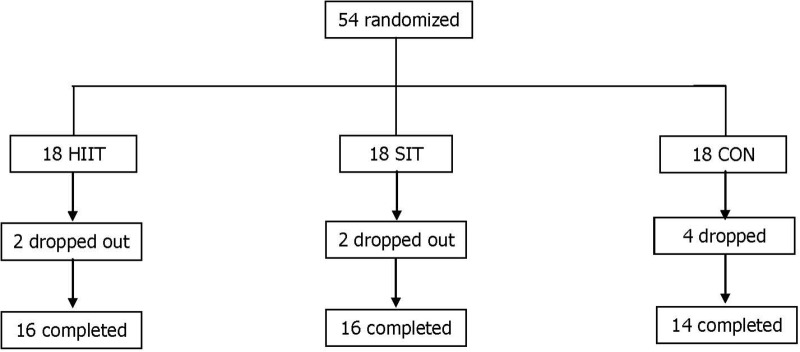
Distribution of study participants. SIT, sprint interval training; HIIT, high-intensity interval training group; CON, control group.

In total, 54 eligible female university students were recruited according to the following inclusion criteria: (1) age range of 18–23 years; (2) body fat percentage ≥30 (as determined through DEXA); (3) body weight remained constant (±2 kg) during the past 3 months; (4) participation in a physical education class twice per week, but not in other regular physical activities or exercise training; and (5) no history of metabolic, hormonal, orthopedic, or cardiovascular diseases, and no current use of prescribed medication including oral contraception. All participants underwent initial assessment and randomization. During the intervention, eight participants did not complete the program for reasons unrelated to the study (**Figure 1**).

Experiments in the present study were performed in accordance with the Helsinki Declaration. Following an explanation of the purpose and constraints of the study, the participants provided written informed consent. The Ethical Committee of Hebei Normal University for the Use of Human and Animal Subjects in Research provided ethical approval of the study.

### Study Design

After pre-intervention assessments, participants with matching body fat percentages were randomly assigned to one of three groups (18 participants in each group): SIT, HIIT, CON. The changes in whole-body and regional abdominal fat, including in the AVFA and ASFA, resulting from these 12 week interventions were subsequently compared among the three groups. All participants were asked to maintain their daily activity and avoid altering their eating habits during the experimental period.

### Exercise Training Protocol

For the SIT protocol, each participant performed a 6 s all-out sprint on a Monark Wingate cycle ergometer (894E, Sweden) followed by a 9 s passive recovery for 80 cycles in a single session. During the last 5 s of each recovery interval participants were given a 5 s countdown and in the last 2 s they were instructed to accelerate with minimum friction applied to the flywheel. At the start of the work interval, the preset load was applied instantaneously with an electromagnetic device. Participants remained seated whilst cycling and their feet were secured to the pedals using toe clips. The cycling load of the SIT was started at 1 kp, The load would be increased by an increment of 0.5 kp whenever the participants demonstrated the adaptation to a given load by completion of the 80 all-out exercise bouts in one session without undue fatigue. The corresponding workload in 1st–4th week was 1.51 ± 0.24 kp; 5th–8th week was 2.95 ± 0.13 kp; 9th–12th week was 3.27 ± 0.25 kp.

The prolonged HIIT protocol was described previously (Zhang et al., 2017). Briefly, participants in each training session repeated 4 min exercise bouts on a cycle ergometer (Monark 839E, Sweden) at an intensity of 90% $\dot{\text{V}}$O_2*max*_, followed by a 3-min passive recovery on the cycle ergometer, until the targeted work was achieved. The pedal frequency during work intervals was maintained at 60 rpm. In the first 2 weeks, the targeted work done was 200 KJ, then increased to 400 KJ gradually during the next 2 weeks, and maintained it during the last 8 weeks. At the end of the 4th and 8th weeks, the $\dot{\text{V}}$O_2*max*_ of all participants was determined to readjust the workload corresponding to a preset intensity. The corresponding workload in 1st–4th week was 2.1 ± 0.3 kp; 5th–8th week was 2.4 ± 0.4 kp; 9th–12th week was 2.5 ± 0.5 kp.

In each training session, the 10 min warm-up and 5 min cool down standardized exercises at 50–60% of HR_*max*_ were identical in SIT and HIIT groups. For the first 4 weeks, the participants in the two experimental groups exercised for one session per day, 3 days per week. During the 5th through 12th weeks, the training frequency was increased to 4 days per week. All participants exercised with close supervision. The exercise HR and perceived physical exertion (Borg RPE scale 6–20) following every five sprints in SIT, and following each exercise bout in HIIT, were recorded. The internal training load of the participants during the SIT and HIIT was quantified by the HR-based method of TRIMP in arbitrary unit (Banister, 1991).

$$\text{TRIMP}=\text{Exercise duration}*(\Delta HR)*0.86*e^{1.672(\Delta HR)}$$

where ΔHR = (HR_*exercise*_ - HR_*rest*_)/(HR_*max*_ - HR_*rest*_), *e* = the base of the Napierian logarithms, and 1.672 is a constant for females. The training adherence of the participants was calculated as the percentage of the actual number of training sessions completed in compliance with the targeted intensity and duration, relative to the total number of training sessions prescribed.

### Body Fat Measurements

Total body mass; body fat percentage; fat mass of the whole-body, trunk, android, and gynoid regions; AVFA; ASFA; and aerobic fitness were measured 1 week before the start of the training program, as well as 3 days after the last training session. During the 2 days of measurements, the participants reported to the laboratory at 8:00 AM after a minimum 8 h fast and refraining from strenuous exercise for 48 h. The sequence of measurements, as follows, was identical pre-intervention and post-intervention.

Body weight and body fat percentage as well as the fat mass of the whole-body, trunk, android, and gynoid regions were measured through DEXA (Discovery Wi, Hologic Inc., Bedford, MA, United States). Regional demarcations were adjusted by a trained technologist according to the guidelines adopted previously (Zhang et al., 2017). Briefly, the trunk region included the area from the bottom of the neckline to the top of the pelvis, excluding the arms; the android region measured from the cut of the pelvic region to 20% of the distance between the pelvic cut and the bottom of the neckline, excluding the arms; and the gynoid region was below the android region and had a height equal to two times that of the android region, with the pelvic cut as the upper demarcation. The ICC between two scans were >0.98, and the output from the DEXA scanner was the fat mass expressed in grams.

The measurements of the abdominal visceral and subcutaneous fat were described previously (Zhang et al., 2017). In details, the cross-sectional AVFA and ASFA were assessed by using a CT scanner (Somatom Definition Flash; Siemens, Forchheim, Germany), with a consistent acquisition protocol set at 120 kVp and 150 mA. During assessment, the participants laid in the supine position with their arms stretched above their heads; a 2 s, 5 mm scan was obtained from the umbilicus level (approximately the L4-L5 intervertebral space). The AVFA and ASFA were evaluated using the built-in volume calculation software of the CT scanner. For each scan, the number of voxels in the entire data set, with CT numbers between −190 and −30 HU, was plotted for adipose tissue. Examining the areas under the curve indicated the total adipose tissue volume. The ICCs for the AVFA and ASFA evaluations between two scans in selected participants were 0.94 and 0.95, respectively.

Identical body fat assessments were performed at the same time of the day pre-intervention and post-intervention, and in avoidance of the menses phases of the participants. The technicians responsible for the DEXA and CT measurements and analyzes were also the same pre-intervention and post-intervention, and were unaware of the participants and intervention groups.

### $\dot{\text{V}}$O_2*max*_ Measurement

The protocol of the graded exercise test conducted on a cycle ergometer (Monark 839E, Sweden) for assessing the $\dot{\text{V}}$O_2*max*_ of the participants was described previously (Zhang et al., 2017). Briefly, the participants began at 50 W with a pedal frequency of 60 rpm; power output was increased by 30 W every 3 min until volitional exhaustion. Oxygen consumption during the exercise test was measured using a Cosmed breath-by-breath metabolic analyzer (Quark-PFT-ergo, Cosmed, Rome, Italy). $\dot{\text{V}}$O_2*max*_ was calculated as the highest 30 s average value. The ICC of the $\dot{\text{V}}$O_2*max*_ measurement in our laboratory was 0.92. Following the graded exercise test, a power output that elicited approximately 90% $\dot{\text{V}}$O_2*max*_ in the HIIT group was selected from the linear relationship of steady-state VO_2_ vs. power output.

### Physical Activity and Dietary Assessments

A diary approach was used to estimate daily energy expenditures according to self-reported physical activity during a 3 week period prior to the intervention and during the 12 week intervention period. The total energy expenditure, expressed as METs.hr.wk^−1^, was estimated on the basis of the METs reported in the Compendium of Physical Activities (Ainsworth et al., 2011) and the duration of recorded activities per week.

The diet (caloric intake) of each participant was recorded on a daily basis during the same periods of the intervention, according to the guidelines of the Sports Nutrition Centre of the National Research Institute of Sports Medicine (NRISM) in China. The dietary records and corresponding energy intake were analyzed each week by a dietician using the NRISM dietary and nutritional analysis system (version 3.1), designed for Chinese athletes and the general population. Dietary advice was provided to the participants by the dietician if the maintenance of the weekly caloric intake was violated.

### Statistical Analysis

The Shapiro-Wilk normality test revealed that all the data for body fat and aerobic fitness were normally distributed. A 2 × 3 two-way ANOVA with repeated measures was used to evaluate the main effects and interactions in the changes of body fat variables across all three groups (SIT, HIIT, CON) between the pre-intervention and post-intervention periods. *Post hoc* analyzes using the Bonferroni test for identifying simple main effects were performed when a significant interaction was detected. A similar ANOVA was used to examine the differences in the habitual energy intake and energy expenditure across all groups between the pre-intervention and intervention periods. For there a significant interaction existed, planned comparisons (paired *t*-tests) with Bonferroni correction were performed to examine the difference between the pre-intervention and intervention periods in each group. All descriptive data were expressed as the mean ± SD. All tests for statistical significance were standardized at an alpha level of *p* ≤ 0.05.

## Results

Of the 54 eligible participants, two participants in SIT and HIIT, and four in CON withdrew from the intervention for personal reasons that were not exercise-related. Accordingly, the data from 16 participants (89%) of the SIT and HIIT, and from 14 participants (78%) of the CON were valid for subsequent analyzes. The age and stature of the participants were not significantly different among the three groups (SIT = 21.3 ± 1.0 years, 161.9 ± 3.7 cm; HIIT = 21.3 ± 1.0 years, 161.6 ± 6.5 cm; CON = 20.7 ± 1.5 years, 161.2 ± 6.5 cm, *p* > 0.05). Among the participants who completed the study, compliance with the exercise intervention was 94 ± 3% and 95 ± 2% in the SIT and HIIT, respectively. No adverse events were reported during testing or training in either group.

The habitual energy intake and energy expenditure of the participants recorded during a 3 week period prior to the intervention and throughout the 12 weeks of the intervention are depicted in **Table 1**. No significant difference was noted within groups between the pre-intervention and intervention periods for habitual energy intake (*p* > 0.05), while both values were higher in HIIT group compared with corresponding values of the other groups (*p* < 0.05). Similar results were also found in the estimated energy expenditure for habitual physical activity, with exercise training being excluded during the intervention period.

**Table 1:** The habitual energy intake and expenditure (excluding training) during the 3-week pre-intervention (Pre) and 12-week intervention (In) periods in SIT, HIIT, and CON groups.

	SIT (*n* = 16)		HIIT (*n* = 16)		CON (*n* = 14)	
	Pre	In	Pre	In	Pre	In
Habitual energy intake (Kcal.d^−1^)	1327 ± 107^α^	1339 ± 107^α^	1985 ± 186	1971 ± 210	1348 ± 341^α^	1342 ± 337^α^
Energy expenditure (METs.hr.wk^−1^)	51.1 ± 6.2^α^	51.1 ± 5.4^α^	77.2 ± 14.9	79.7 ± 15.7	55.4 ± 16.1^α^	54.4 ± 14.5^α^

*Values are expressed as means ± standard deviation. ^α^Significantly different from the corresponding HIIT values (p < 0.05); The differences between Pre and In in each variable were not significant in all groups (p > 0.05).*

During the SIT, all participants were able to complete the protocol of 80 × 6 s sprints in each training session. The accumulated exercise time of 8 min for each session of SIT during the whole intervention was significant shorter than that of HIIT in 1–4 weeks (27.6 ± 2.9 min), as well as in 5–8 (48.2 ± 6.4 min), and 9–12 (46.3 ± 6.3 min) weeks (*p* < 0.05). Other details of the exercise in the training sessions of SIT and HIIT are shown in **Table 2**. During the intervention, the exercise intensity revealed by the mean power output increased progressively in both groups (*p* < 0.05), and was significantly higher in SIT by comparing with that in HIIT (*p* < 0.05). However, the corresponding exercise HR in SIT was similar to or a little lower than that in HIIT (*p* < 0.05). The average RPE was significant lower in SIT throughout the intervention (*p* < 0.05). For the training load, the TRIMP of SIT was maintained throughout the intervention, and was significant lower than that of HIIT (*p* < 0.05). The TRIMP of HIIT was increased significantly following 1–4 weeks when the participants were encouraged to increase the work done to 400 KJ in 5–12 weeks (*p* < 0.05).

**Table 2:** The power output, HR, and rating of perceived exertion (RPE) of training sessions in every 4 weeks during the 12 week intervention in HIIT and SIT groups.

	SIT(*n* = 16)			HIIT (*n* = 16)		
	Week 1–4	Week 5–8	Week 9–12	Week 1–4	Week 5–8	Week 9–12
Power output (W)	203.0 ± 18.3^α^	238.5 ± 16.7^αβ^	270.3 ± 25.7^αβ^	123.1 ± 19.8	141.7 ± 26.0^β^	147.1 ± 27.0^β^
HR (beats.min^−1^)	165.0 ± 9.1^α^	164.6 ± 5.9	164.6 ± 6.2	173.7 ± 8.8	169.8 ± 7.7	164.5 ± 8.6^β^
RPE	14.4 ± 1.1^α^	14.7 ± 0.8^α^	14.9 ± 0.9^α^	16.7 ± 0.9	17.2 ± 0.6	17.1 ± 0.5
TRIMP (au)	56.4 ± 11.4^α^	56.5 ± 7.1^α^	56.7 ± 8.3^α^	143.6 ± 35.6	241.2 ± 50.7^β^	220.9 ± 43.3^β^

*Values are expressed as means ± standard deviation. TRIMP (au): training impulse (arbitrary unit). ^α^Significantly different from the corresponding values of HIIT (p < 0.05). ^β^Significantly different from the corresponding values of week 1–4 (p < 0.05).*

The pre- and post-intervention body mass, body fat percentage, whole-body fat mass and the fat mass of the android, gynoid, and trunk regions of all three groups, as well as the repeated measures ANOVA results, are shown in **Table 3**. The baseline total body mass and body fat percentage exhibited no significant difference among the three groups (*p* > 0.05). After the 12week intervention, a significant reduction in body mass and body fat percentage was observed in SIT and HIIT (*p* < 0.05), but not in CON. Although the reduction in body mass in SIT was relatively less than that in HIIT (*p* < 0.05), the reduction in body fat percentage did not differ significantly between the two groups (*p* > 0.05).

**Table 3:** Pre- and post-intervention levels and changes in body mass, % body fat, fat mass (FM) of whole-body, and regions of android, gynoid and trunk, as well as abdominal visceral (AVFA) and subcutaneous (ASFA) fat areas in SIT, HIIT, and CON groups.

	SIT (*n* = 16)		HIIT (*n* = 16)		CON (*n* = 14)		ANOVA p value (group, time, interaction)
	Pre	Post	Pre	Post	Pre	Post	
Body mass (kg)	66.7 ± 6.4	64.9 ± 6.2^∗∗^	68.9 ± 12.1	64.9 ± 10.2^∗∗^	68.2 ± 9.9	67.8 ± 10.4	0.81, 1 × 10^−4^, 1 × 10^−4^
	[-1.7(-2.8, -0.6)]^α^		[-4.0(-5.5, -2.5)]^††^		[-0.4(-1.7, 0.7)]		

% body fat (%)	38.4 ± 2.3	38.2 ± 2.4	38.2 ± 2.4	38.2 ± 2.4	40.5 ± 2.6	40.5 ± 3.7	1 × 10^−4^, 1 × 10^−4^, 1 × 10^−4^
	[-2.0(-2.8, -1.4)]^††^		[-1.9(-2.7, -1.1)]^†^		[0.1(-1.3, 1.4)]		

Whole-body FM (kg)	25.7 ± 3.5	23.7 ± 3.3^∗∗^	26.6 ± 6.1	23.8 ± 5.0^∗∗^	27.9 ± 5.1	27.4 ± 5.9	0.20, 1 × 10^−4^, 1 × 10^−4^
	[-2.0(-2.7, -1.3)]^†^		[-2.8(-3.8, -1.8)]^††^		[-0.4(-1.7, 0.9)]		

Andriod FM (kg)	2.0 ± 0.4	1.8 ± 0.4^∗∗^	2.1 ± 0.6	1.9 ± 0.6^∗∗^	2.2 ± 0.5	2.1 ± 0.6	0.29, 1 × 10^−4^, 0.61
	[-0.2(-0.3, -0.1)]		[-0.2(-0.3, -0.1)]		[-0.1(-0.2, 0.0)]		

Gynoid FM (kg)	4.6 ± 0.6	4.2 ± 0.6^∗∗^	4.6 ± 0.8	4.3 ± 0.7^∗∗^	5.0 ± 1.0	4.9 ± 1.0	0.17, 1 × 10^−4^, 3 × 10^−2^
	[-0.4(-0.6, -0.3)]^†^		[-0.3(-0.4, -0.2)]		[-0.1(-0.4, 0.1)]		

Trunk FM (kg)	11.5 ± 2.2	10.3 ± 2.0^∗∗^	12.7 ± 3.6	10.7 ± 2.8^∗∗^	12.7 ± 2.2	12.5 ± 3.0	0.22, 1 × 10^−4^, 1 × 10^−4^
	[-1.2(-1.6, -0.7)]^†α^		[-2.0(-2.6, -1.3)]^††^		-0.1(-1.1, 0.8)		

AVFA (cm^2^)	69.3 ± 23.4	62.9 ± 21.9^∗^	69.1 ± 32.8	59.3 ± 23.9^∗∗^	67.6 ± 19.9	67.4 ± 20.9	0.9, 1 × 10^−4^, 3 × 10^−2^
	[-6.31(-11.4, -1.2)]^†^		[-9.7(-16.3, -3.1)]^†^		[-0.2(-3.1, 2.6)]		

ASFA (cm^2^)	244.9 ± 62.3	227.5 ± 51.8^∗^	263.2 ± 84.1	222.5 ± 71.3^∗∗^	276.8 ± 78.1	275.2 ± 77.3	0.27, 1 × 10^−4^, 1 × 10^−4^
	[-17.4(-33.8, -0.9)]^α^		[-40.7(-55.2, -26.2)]^††^		[-1.5(-14.6, 11.5)]		

AVFA+ASFA (cm^2^)	314.2 ± 77.5	290.5 ± 69.0^∗^	332.3 ± 110.1	281.8 ± 89.8^∗∗^	344.4 ± 84.6	342.6 ± 84.3	0.37, 1 × 10^−4^, 1 × 10^−4^
	[-23.7(-42.8, -4.5)]^α^		[-50.5(-69.1, -31.8)]^††^		[-1.8(-16.6, 13.1)]		

*Values are expressed as means ± standard deviation. ^∗^Significantly different from the corresponding Pre values (p < 0.05), ^∗∗^ (p < 0.01). ^†^Significantly different from the corresponding CON values (p < 0.05), ^††^(p < 0.01). ^α^Significantly different from the corresponding HIIT values (p < 0.05).*

For the whole-body fat mass and the fat mass of the android, gynoid, and trunk regions, the baseline values did not differ significantly among the three groups (*p* > 0.05). Subsequent to the 12 week intervention, significant reductions in all variables were noted among both the SIT and HIIT (*p* < 0.05); CON had no changes. The reductions in whole-body and regional fat mass did not vary between the two intervention groups (*p* > 0.05), except the reduction in trunk fat mass in SIT which was less compared with that in HIIT (*p* < 0.05).

**FIGURE 2:**
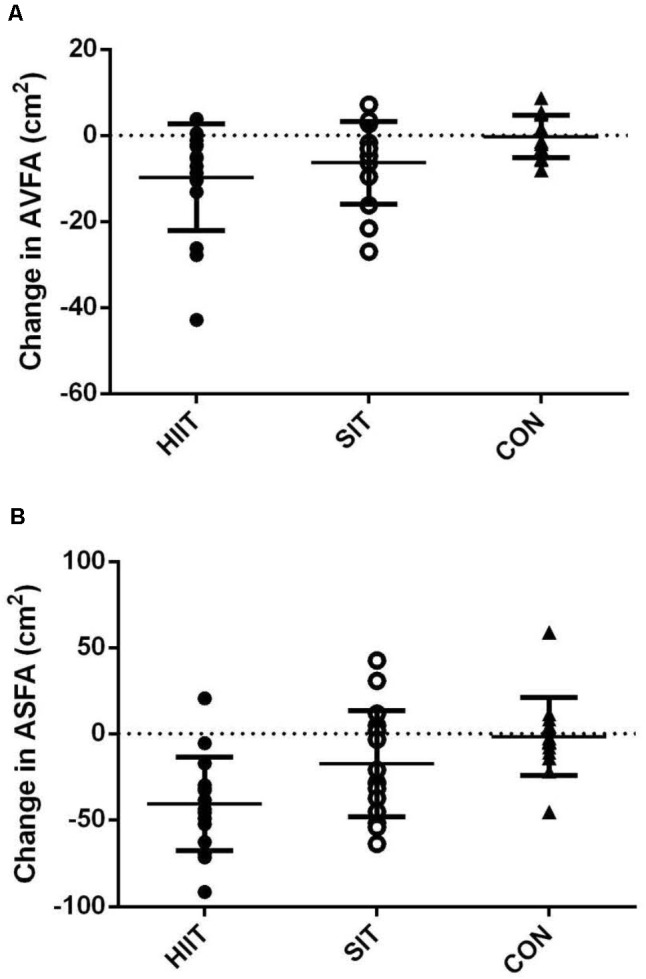
Changes in **(A)** AVFA, and **(B)** ASFA of participants post-intervention in HIIT, SIT, and CON groups.

**Table 3** also shows the pre- and post-intervention AVFA, ASFA, and combined AVFA and ASFA of all groups, as well as the repeated measures ANOVA results. The baseline variables did not differ significantly among the three groups (*p* > 0.05). Following the 12 week intervention, both AVFA and ASFA reduced significantly in SIT and HIIT. The reduction in AVFA between the two intervention groups was not different (*p* > 0.05, **Figure 2**). However, the reductions in ASFA and the combined ASFA and AVFA in the SIT were significant less in comparison to those in HIIT (*p* < 0.05). CON had no change in all variables (*p* > 0.05).

Following the 12 week interventions, $\dot{\text{V}}$O_2*max*_ increased significantly in the SIT (Pre: 30.7 ± 3.5, Post: 38.5 ± 4.8 mL.kg^−1^.min^−1^) and HIIT (Pre: 30.2 ± 4.4, Post: 34.3 ± 4.6 mL.kg^−1^.min^−1^, *p* < 0.01), but not in the CON (Pre: 28.6 ± 3.1, Post: 28.0 ± 3.0 mL.kg^−1^.min^−1^, *p* > 0.05). The increase in $\dot{\text{V}}$O_2*max*_ in SIT was significant greater than that in HIIT group (*p* < 0.01).

## Discussion

Our previous studies (Zhang et al., 2015, 2017) have demonstrated the time-efficient characteristics of the HIIT in the body fat reduction, mainly the portions of abdominal visceral and subcutaneous, by comparing with moderate-intensity continuous training. The present study intended to be more ambitious in scope as compared to previous HIIT studies by involving an “all-out” SIT regime with relative higher training intensity, and less training load and volume, and an explicit prolonged HIIT protocol. The major novel findings of this study were that 12 week SIT in obese young women reduced whole-body and most regional fat mass, as well as AVFA, to the same extent as that resulting from the HIIT despite a significant lower training load (2.5–4.3-fold) and exercise time (3.5–6-fold) commitment in each session. Moreover, SIT elicited a more desirable aerobic adaptation. In addition of the reasonable feasibility of the SIT regime in obese young women revealed by the high compliance of the participants with the exercise intervention, it appears that the SIT regime could optimize the time-efficiency advantage of the traditional HIIT in developing a habitual strategic exercise for combatting obesity.

In the present study, although the exercise intensity based on the cycling power output (**Table 2**) was somewhat higher in SIT, the training load revealed by the TRIMP (**Table 2**) was fourfold higher in the HIIT. Accordingly, the associated energy expenditure for repeating the high-intensity exercise during the prolonged HIIT sessions, in addition of the subsequent post-exercise metabolic rate were reasonably deemed to be comparable to, or possibly higher than that of SIT. During the SIT and HIIT, the energy metabolism for the high-intensity exercise intervals mainly relied on carbohydrates as a substrate; while fatty acid mobilization from adipose tissue was the dominant fuel source for the elevated post-exercise metabolism (Romijn et al., 1993). The potential higher energy expenditure in HIIT might have led to more fat loss found in trunk region as well as the subcutaneous portion in abdomen (**Table 3**), but it did not concomitantly result in distinct greater reduction in visceral fat notwithstanding (**Figure 2**). The sluggish response in the visceral fat reduction to the high training volume of HIIT was further revealed when the change in AVFA (-9.7 cm^2^) of our participants was compared to that (-9.1 cm^2^) of age-matched, obese female participants following a 12 week HIIT with relative lower cycling work done (300 KJ) in our previous study (Zhang et al., 2017). Such findings corroborate the lack of dose-response relationship between training volume and visceral fat reduction resulting from HIIT in metabolically healthy individuals that have been reported previously (Logan et al., 2016; Zhang et al., 2017). It further implies that the exercise intensity, rather than the training volume, is more likely to be a relevant variable for modifying visceral fat storage by means of interval training in young obese women.

However, our current data could not explain clearly the underlying mechanism for the comparable visceral fat loss in the SIT and HIIT. Potentially this endeavor might harness the benefits of the higher exercise intensity in SIT and associated higher secretion of lipolytic hormones (Zhang et al., 2017), along with the greater primary motor cortex activity in response to the repeated-sprint maneuver (Kjaer et al., 1996). Catecholamines and growth hormones are lipolytic hormones which increase demonstrably with exercise intensity (Pritzlaff et al., 2000; Zouhal et al., 2013), despite of the blunted catecholamine responses to repeated cycling sprints have been reported in obese individuals (Jabbour et al., 2011). The possible higher catecholamine secretion resulting from the greater sympathetic activation in response to the higher exercise intensity in the SIT might have driven lipolysis from visceral fat storage via β_3_-adrenoceptors (Zouhal et al., 2013; Maillard et al., 2018) in a greater extent in comparison to that of HIIT. Moreover, the greater central motor activity (Kjaer et al., 1996) in accomplishing the maximum power output in a repeated, transient “start-stop” manner in SIT, along with the associated repeating stimulations in various signaling pathways including adrenergic pathway (Weltman et al., 2000; Gibala and Hawley, 2017) might have potentiated the exercise-induced growth hormone release, facilitating a reduction of visceral fat (Freda et al., 2008). The elevated exercise-induced lipolytic hormones in plasma which have been shown to sustain for more than an hour after a brief session of high-intensity interval exercise, might have also contributed to the specific fat loss in association to the post-exercise fat metabolism (Williams et al., 2013; Sasaki et al., 2014).

However, despite the exercise-induced increase in plasma catecholamines is in a dose-response relationship with exercise intensity, the corresponding increase in rate of lipolysis was reported to be out of phase; becoming plateau and decrease when the exercise intensity and associated plasma catecholamines approach to maximum (Bartlett et al., 2010). The high-level blood lactate during heavy exercise have been shown to mitigate the lipolytic effects of catecholamines by promoting triglyceride esterification with the presence of α glycerol phosphate (Romijn et al., 1993). The associated elevated H^+^ may also inhibit the hormone sensitive lipase involved in triglyceride breakdown into FFA and glycerol (Fain and Shepherd, 1976). Moreover, reduction in blood flow through the adipose tissues due to sympathetic vasoconstriction in response to the high-intensity exercise may result in less mobilization of fat (Romijn et al., 1993). Such physiological responses to heavy exercise imply that the extreme high intensity of the repeated all-out supramaximal sprint intervals during SIT might have not mediated optimal visceral fat modification in the participants. Besides, the elevations of growth hormone in response to a single 6 s sprint was found to be in a lesser magnitude in comparing to that resulting from 30 s sprint (Stokes et al., 2002). Further, there were evidences that running HIIT-induced visceral fat reduction is potentially more effective than that of cycling HIIT (Maillard et al., 2018). The relative small amount of AVFA reduction (6.31 cm^2^) subsequent to the 12 week SIT in the present study in comparison to that (11.8 cm^2^) following 12 week interval training composed of four 4 min high-intensity intermittent run in our previous study (Zhang et al., 2015) might have hinted the potential roles of the duration of exercise intervals and exercise modality account for the exercise-induced visceral fat loss. These come up with an uncertainty of whether the current SIT regime accomplished by repeating numerous “all-out” 6 s sprints on cycle ergometer favors the efficient visceral fat lipolysis in obese individuals. Nevertheless, the current study provides explicit evidences that the SIT regime could induce a more desirable aerobic adaptation in comparison to that of HIIT. Such findings support the previous notion that the duration or frequency of interval training appear to have smaller effects, compared to that of exercise intensity, in mediating improvements in $\dot{\text{V}}$O_2*max*_ (MacInnis and Gibala, 2017).

### Limitations

Apart from the aforementioned potential limitations of the current SIT regimen and exercise modality in demonstrating the optima of the specific training in reducing abdominal visceral fat, another potential limitation of the present study is the lack of precise measures to ensure the participants to give “all-out” effort in every single sprint during the SIT. Nevertheless, the all-out effort of the participants in the SIT group was partially revealed by the higher mean power output relative to that of HIIT in all the stages of the 12 week intervention (**Table 2**). In fact, it has been reported that untrained individuals were not likely to adopt a pacing strategy unconsciously during all-out repeated sprint cycles when the duration of a single exercise bout is no more than 15 s (Wittekind et al., 2011). Besides, we recognize the individual differences in the visceral fat reduction response to both SIT and HIIT (**Figure 2**). The variations may be partly attributed to the gene polymorphisms which account for the inter-individual variability in response to exercise training (Mori et al., 2009). As there are likely to be fat-loss responders and non-responders in the SIT and HIIT groups, the effectiveness of either training regimen revealed by calculating the mean fat loss alone may hide the significance of fat loss achieved by some individuals, while over-estimate it in others. Thus, caution is needed in the interpretation of the effectiveness of either regimen on producing clinical decrease in visceral fat in general populations.

### Future Directions

It is general agreed that the high-intensity exercise sessions of HIIT, rather than the training volume, dominates the function of the training regimen in eliminating excessive visceral fat. However, the minimal dose of HIIE for the maximum health benefit is still being identified. As discussed earlier, the physiological responses elicited by the repeated all-out supramaximal sprints in SIT may not appear entirely favorable to the mobilization of both visceral and subcutaneous fat. To investigate the optimal time-efficient interval training regimen by adoptions of lighter training intensity and lower training volume (lesser repetitions per session, etc.), along with longer interval duration, and altered exercise modality for combatting obesity-related complications are of interest. Moreover, training-induced visceral fat reduction are more manifest in men (Vissers et al., 2013), future investigations on the visceral fat reduction with reformed training regimens should be gender-specific. Lastly, although HIIT appears to be both safe and beneficial for the patients and older adults with chronic disease and increased risk for exercise-related complications (Boutcher, 2011), evidence is limited on the feasibility of the SIT in these special populations. Comprehensive studies for establishing the most beneficial SIT regimen that is optimal and sustainable for different populations are warranted in this regard.

## Conclusion

Both 12 week SIT and HIIT regimens successfully produced significant reductions in the fat mass of the whole-body and the android, gynoid, and trunk regions, as well as of the AVFAs and ASFAs in obese young females. The magnitude of the fat reduction for each category in SIT was mostly comparable to that of HIIT, except less reduction was found in the ASFAs and the fat mass in the trunk region. Moreover, SIT mediated more desirable improvements in $\dot{\text{V}}$O_2*max*_ in the participants. Such findings imply that the SIT regimen, which demands less commitments from participants in training load and exercise time, may further optimize the time-efficiency advantage of the traditional HIIT in body fat regulation and aerobic fitness advancement.

## Author Contributions

HZ and TT contributed to research the design. HZ, HS, JA, and YL collected the data. HZ, TT, JN, and ZK analyzed and interpreted the data. TT and HZ drafted the manuscript. TT, HZ, and JN revised the manuscript.

## Conflict of Interest Statement

The authors declare that the research was conducted in the absence of any commercial or financial relationships that could be construed as a potential conflict of interest.
